# The ciliopathy protein TALPID3/KIAA0586 acts upstream of Rab8 activation in zebrafish photoreceptor outer segment formation and maintenance

**DOI:** 10.1038/s41598-018-20489-9

**Published:** 2018-02-02

**Authors:** Irene Ojeda Naharros, Flavia B. Cristian, Jingjing Zang, Matthias Gesemann, Philip W. Ingham, Stephan C. F. Neuhauss, Ruxandra Bachmann-Gagescu

**Affiliations:** 1 Institute for Molecular Life Sciences, University of Zurich, 8057 Zurich, Switzerland; 2 Lee Kong Chian School of Medicine, Nanyang Technological University, 639798 Singapore, Singapore; 3 Institute for Medical Genetics, University of Zurich, 8952 Schlieren, Switzerland; 4 Present Address: Department of Human Molecular Genetics, Institute of Human Genetics, University of Heidelberg, Heidelberg, Germany

## Abstract

Ciliopathies are human disorders caused by dysfunction of primary cilia, ubiquitous microtubule-based organelles involved in signal transduction. Cilia are anchored inside the cell through basal bodies (BBs), modified centrioles also acting as microtubule-organization centers. Photoreceptors (PRs) are sensory neurons, whose primary cilium forms a highly specialized compartment called the outer segment (OS) responsible for sensing incoming light. Thus, ciliopathies often present with retinal degeneration. Mutations in *KIAA0586*/*TALPID3* (*TA3*) cause Joubert syndrome, in which 30% of affected individuals develop retinal involvement. To elucidate the function of TALPID3 in PRs, we studied *talpid3* zebrafish mutants and identified a progressive retinal degeneration phenotype. The majority of PRs lack OS development due to defects in BB positioning and docking at the apical cell surface. Intracellular accumulation of the photopigment opsin leads to PR cell death of moderate severity. Electroretinograms demonstrate severe visual impairement. A small subset of PRs display normally docked BBs and extended OSs through rescue by maternally-deposited Talpid3. While localization of the small GTPase Rab8a, which plays an important role in BB docking, appears unaffected in *talpid3−/−* PRs, overexpression of constitutively active Rab8a rescues OS formation, indicating that the role of Ta3 in early ciliogenesis lies upstream of Rab8a activation in PRs.

## Introduction

Ciliopathies are a group of human disorders caused by primary cilium dysfunction and unified by a wide array of overlapping phenotypes including central nervous system malformations, kidney cysts or retinal degeneration^1–3^. Primary cilia are ubiquitous organelles that consist of a mother centriole-derived basal body (BB), a microtubule-based axoneme and a specialized membrane that harbors proteins required for signal detection^4^. Indeed, the main function of primary cilia lies in transduction of a wide range of extracellular signals, including important morphogens such as Hedgehog (Hh)^3^ or environmental stimuli such as light^5,6^. Light transduction is carried out by retinal photoreceptors (PRs), which are highly polarized sensory neurons with a synaptic terminal, a cell body, an inner segment (IS) and a modified primary cilium called the outer segment (OS)^7,8^. The OS consists of stacks of membranous disks which are organized around a microtubule-based axoneme and which contain proteins required for phototransduction, such as the photopigment opsin^9^. The connecting cilium, which is partly equivalent to the transition zone in other cilia types, joins the OS with the IS^7^.

Mutations in *KIAA0586* lead to Joubert syndrome (OMIM 213300)^10,11^, a canonical ciliopathy characterized by a pathognomonic hindbrain malformation^12–14^ and associated with retinal dystrophy in 30% of individuals^15^. More severe ciliopathies with fetal lethality such as hydrolethalus syndrome (OMIM 236680), consisting of major hydrocephaly and brain malformations, and short-rib polydactyly (OMIM 616546), including skeletal dysplasia, have also been associated with causal *KIAA0586* mutations^16^.

The *KIAA0586/TALPID3* (*TA3*) gene was originally identified in a spontaneous chicken mutant displaying phenotypes characteristic of defective Hh signaling: craniofacial malformations, left-right asymmetry defects, polydactyly and neural tube mispatterning^17,18^. These Hh defects were subsequently shown to be secondary to a defect in ciliogenesis^19^, since Hh cannot be transduced properly in the absence of a functional primary cilium^3^. Indeed, a role for Ta3 in early ciliogenesis steps (BB-migration and docking) was identified in neural tube cells of a *Ta3* conditional knock-out mouse model^20^ and in ependymal cells in the chick mutant^21^. Consistent with these findings, cell culture studies revealed that Ta3 localizes to a rim crowning both the mother and daughter centrioles in RPE1 cells, and that it plays an essential role in early ciliogenesis including centriolar satellite dispersal and recruitment of the small GTPase Rab8 required for BB docking^22^. In addition, work in the chicken suggested a role for Talpid3 in cell and tissue polarity^23^. In the same study, Talpid3 was also shown to localize to the base of PR cilia in mice and humans^23^, but its function in PRs has not been investigated so far. Given that PR primary cilia are so highly specialized, it remains indeed an open question, whether dysfunction of ciliopathy proteins leads to retinal dysfunction through the same mechanisms as those affecting other cell types with more “canonical” primary cilia.

To investigate the role of TA3 in retinal photoreceptors, we studied the retina of *ta3* mutant zebrafish. Using a custom-developed anti-zebrafish-Ta3 antibody, we confirmed zebrafish Ta3 localization to both mother and daughter centrioles in zebrafish PRs. We further identified retinal dystrophy as a novel phenotype linked to *ta3* dysfunction: retinal lamination and initial PR differentiation proceeded normally but were followed by progressive PR cell death. Intracellular opsin accumulation was observed in the majority of PRs, along with lack of OS formation. Abnormal BB positioning and deficient docking at the apical membrane underlied the OS development defect. Finally, while we observed no substantial defects in Rab8a localization in our model, overexpression of a constitutively active form of the small GTPase Rab8a rescued OS formation. These results indicate that Ta3, through its function in early ciliogenesis, plays a crucial role in PR development and function and that this role lies upstream of Rab8a activation.

## Results

### Ta3 localizes to mother and daughter centrioles of zebrafish PR primary cilia and is lost from the majority of PRs in zygotic *ta3* mutants

**Figure 1:**
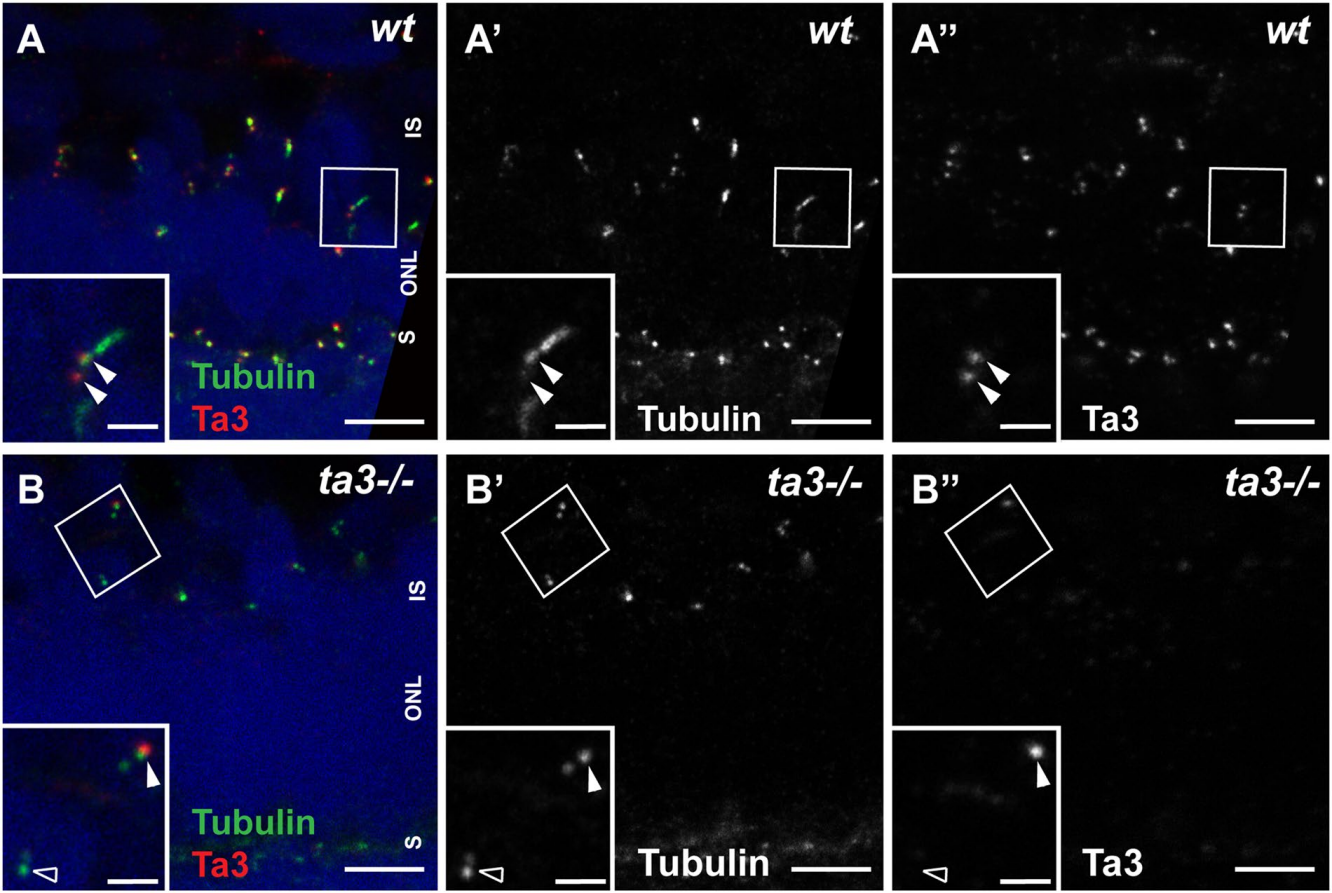
Ta3 localizes to mother and daughter centrioles of photoreceptor primary cilia and is mostly absent in zygotic *ta3* mutant PRs. (**A–A”**) 3 dpf retinal cryosections stained with anti-acetylated tubulin to mark the nascent cilium (green) and with anti-Ta3 (red) show localization of Talpid3 at the base of PR cilia in wildtype (wt) larvae. The double arrowheads highlight Ta3 localization at mother and daughter centriole. (**B–B”**) In 3 dpf cryosections from *ta3−/−* larvae, the Ta3 signal is mostly abolished (empty arrowhead in (**B”**)), but isolated BBs still have positive Ta3 signal, likely maternally deposited (arrowhead in (**B”**)). The boxed areas in (**A–B”**) are shown as an inset at the bottom left of each corresponding panel. ONL outer nuclear layer, OS outer segment, S synapse. Scale bars: 2.5 µm in (**A**–**B”**), 1 µm in insets.

Previous work on zebrafish *talpid3* (*ta3*) demonstrated strong ubiquitous RNA expression in the early zebrafish embryo and at 2 days post fertilization (dpf)^24^. To determine *ta3* expression pattern and subcellular localization in developing zebrafish photoreceptors (PRs) at later stages, we developed a polyclonal antibody directed against the zebrafish Ta3 protein. In wildtype zebrafish, Ta3 was found to co-localize with Centrin in PRs from 2 dpf onwards (Fig. S1) and to be present at the base of the cilium highlighted with anti-acetylated tubulin antibody at 3 (Fig. 1) and 4 dpf (Fig. S1). Endogenous Ta3 protein was detected in both mother and daughter centrioles in retinal PRs as previously reported for an EGFP-Ta3 fusion protein in zebrafish^24^, in cultured hTERT-RPE1 cells and in mouse and human PRs^22^.

To investigate the role of Ta3 in retinal PRs, we studied three previously generated mutant *ta3* alleles: *i262, i263* and *i264*, each of which harbours a small deletion or insertion leading to frameshifts in exon 11, encoding part of the fourth coiled-coil domain^24^. Previously reported phenotypes of these zebrafish *ta3* mutants included curved body shape, mild cyclopia and left-right asymmetry disruption in maternal-zygotic (mz) mutants. In contrast, zygotic *ta3* mutants displayed a milder phenotype, with apparently isolated cystic kidneys, likely due to maternally deposited Ta3 partially rescuing earlier phenotypes^24^. The phenotypes of the three zygotic mutants being indistinguishable from each other, they will be referred to hereafter as *ta3−/−* or *ta3* mutants. Using our custom-developed anti-zebrafish-Ta3 antibody, we found that Ta3 signal was lost from almost 85% of BBs in PRs of zygotic *ta3* mutants (n = 13/78 tubulin-marked BBs were positive for Ta3 signal in 3dpf *ta3−/−* larvae compared to 74/78 in wild-type larvae; 4 wt and 4 mutant larvae), supporting specificity of the antibody and indicating that the vast majority of *ta3−/−* PRs lack Ta3 protein at 3 dpf (Fig. 1B–B” and Fig. S1). However, we observed persistent Ta3 staining in a few isolated mutant PRs up to 4 dpf (Fig. S1) and in these cells, extended axonemes were present as highlighted with anti-acetylated Tubulin staining. Together, these results indicate that Ta3 is located at both centrioles in zebrafish PRs, that it is mostly absent from PRs of zygotic *ta3* mutants but that isolated PRs in these mutants retain maternally-derived Ta3.

### Zygotic *ta3−/−* larvae show progressive photoreceptor degeneration

**Figure 2:**
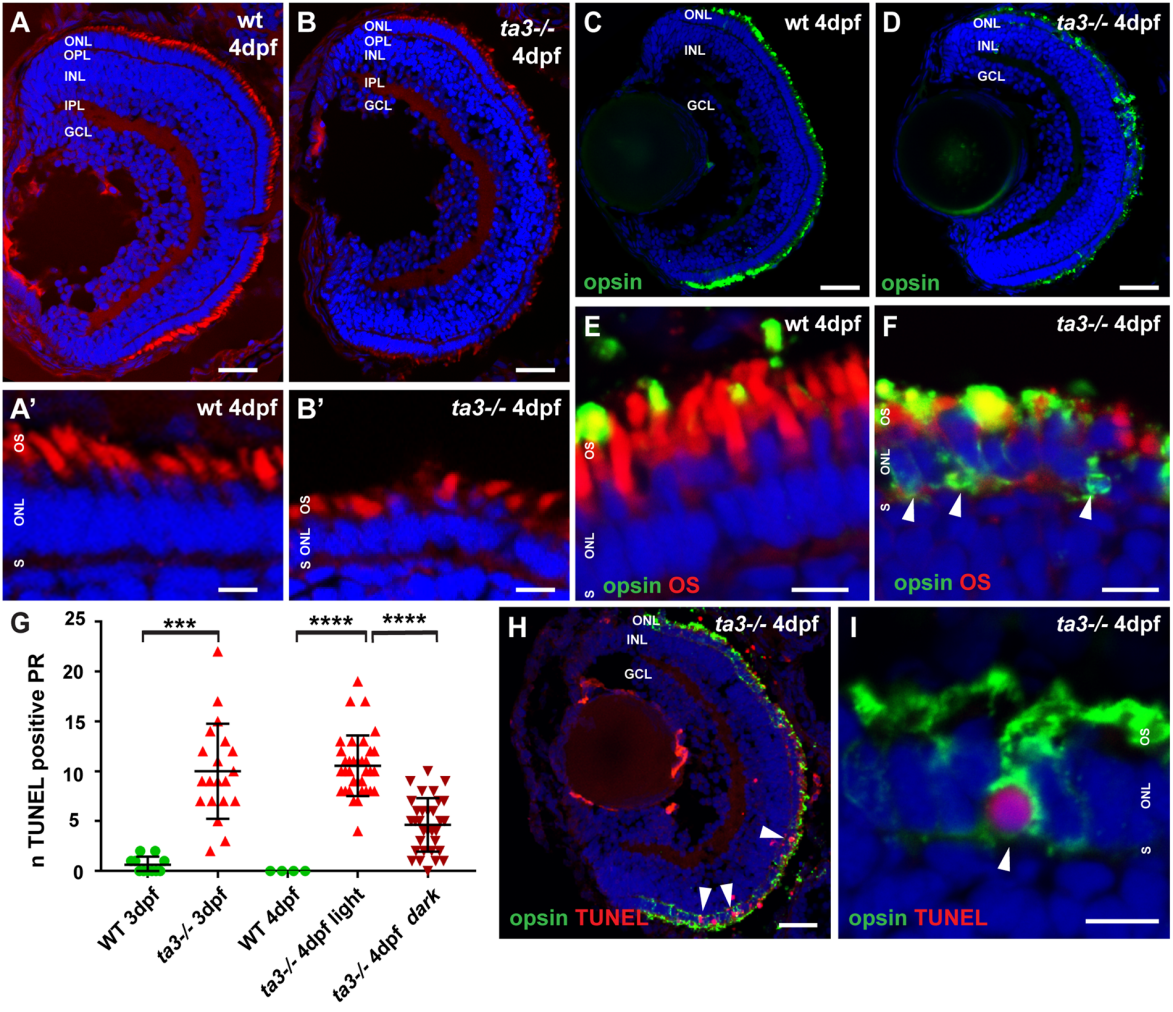
t*a3* mutants show progressive retinal degeneration and intracellular opsin accumulation. (**A**,**B**) Retinal lamination is unaffected in *ta3* mutants as seen on cryosections at 4 dpf stained with the lipophilic dye BODIPY (red) to mark cell membranes and outer segments and DAPI (blue) to mark nuclei. Note the reduced number of PRs with OSs and the cell shape changes in *ta3* mutants compared to wildtype (wt) (**A’–B’**). (**C,D**) Marked intracellular opsin accumulation on 4 dpf cryosections stained with 4D2 antibody (green) recognizing rhodopsin and red-green cone opsin on whole eye cryosections. Nuclei are counterstained with DAPI. (**E**,**F**) High magnification images of the PR cell layer of wt (**E**) and *ta3* mutant (**F**) cryosections at 4 dpf stained with 4D2 antibody (green in **E,F**) and with BODIPY (red in **E,F**) to highlight the OSs. Note the substantial intracellular opsin accumulation in mutant PRs in (**F**) (arrowheads). (**G**) Progressive PR cell death in *ta3* mutants as assessed with a TUNEL assay. Note the significantly smaller amount of TUNEL positive cells in 4 dpf *ta3−/−* larvae raised in darkness (dark red inverted triangles) compared to those raised in a normal light cycle (light red triangles). Quantification was performed on confocal stacks of identical thickness from cryosections of whole retinas through equivalent regions of the eyes. Each datapoint indicates the number of TUNEL positive nuclei counted in a single larva. ***p < 0.001, ****p < 0.0001, Student’s *t*-test. Bars represent standard deviation. (**H**) Whole *ta3−/−* eye cryosection stained with TUNEL assay (red) and with 4D2 antibody (green). Arrowheads mark TUNEL positive PRs. (**I**) Higher magnification image of a PR with substantial opsin mislocalization (4D2, green) and positive TUNEL reaction (red, arrowhead). *dpf* days post fertilization, *GCL* granule cell layer, *INL* inner nuclear layer, *IPL* inner plexiform layer, *n* number, *NS* not significant, *ONL* outer nuclear layer, *OPL* outer plexiform layer, *OS* outer segment, *PR* photoreceptor, *S* synapse, *wt* wild-type. Scale bars: 30 µm in (**A**,**B**) and H, 4 µm in (**A’**–**B’**), (**E**,**F**) and I, 40 µm in (**C**,**D**).

Initial stages of eye development up to 2 dpf were unaffected in *ta3* mutants, with normal retinal lamination (Figs 2A,B and S2A–J) and formation of the PR cell layer. Developing cells in this layer were able to adopt PR-subtype specific cell fates as seen by zpr1 (Fret43) antibody staining (Fig. S3A,B’), to mark red-green cones^25^ and with the *tg(zfRH1-3.7B:EGFP)* transgenic line that highlights rods^26^ (Fig. S3C–D’). At 3 dpf however, PR cell death became apparent as seen with TUNEL assay and persisted up to the latest analyzed stage at 5dpf (Figs 2G and S4). We compared the extent of cell death to that observed in the ciliary mutant *oval*, which harbours a nonsense mutation in the intraflagellar transport gene *ift88* that causes substantial PR cell death^27,28^, leading to quasi-absence of PRs in the central retina by 5 dpf. Compared to *oval* mutants, *ta3* mutant retinae showed a similar extent of PR cell death at 3 dpf, but slightly less severe cell death at 4 dpf (Fig. S4). Consequently, PRs were still present in the central retina at 5 dpf and beyond in *ta3* mutants, despite a thinning of the PR cell layer (Fig. S2). Together, these data indicate that PRs start to differentiate normally in *ta3* mutants, taking on PR-subtype specific cell fates, but that moderate PR cell death occurs starting at 3 dpf, leading to a reduction in the number of PRs.

### Marked intracellular opsin mislocalization in *ta3−/−* PRs

PR cell death has been shown to be secondary to intracellular opsin accumulation in many ciliary mutants such as *Kif3a−/−* or *Spata1−/−* mice, as opsin accumulation preceded cell death and additional knock-out of Rhodopsin in these mice (*Kif3a−/−*; *Rho−/−* and *Spata1−/−*; *Rho−/−* double mutants) partially rescued the PR cell death phenotype^29,30^. We therefore determined whether opsins accumulate intracellularly in *ta3* mutant photoreceptors by immunofluorescence using antibodies against opsin (4D2 antibody recognizing rhodopsin and red-green cone opsin). We observed significant intracellular accumulation of opsins in *ta3−/−* rods and cones at 4 dpf (Fig. 2C–F). This intracellular opsin accumulation was observed at 3 dpf already and persisted until 6 dpf, the latest stage analysed. Substantial variability was observed in the amount of intracellular opsin accumulation even between PRs in the same retina, with a minority of PRs demonstrating normal opsin localization to the OS, while strong signal was mainly detected in the cell body of other PRs. The precise mechanism by which intracellularly accumulated opsins lead to cell death still requires a definitive explanation; while activation of the photopigment was suggested to be required for causing cell death^31^, other studies found no difference in PR cell death rates in mice raised in constant darkness compared to those raised in normal light conditions and no requirement for signaling from an opsin-arrestin complex or for photoactivation^29,30^. To test the relationship between intracellular opsin accumulation and PR cell death in zebrafish *ta3* mutants, we performed a TUNEL assay together with anti-opsin immuno-fluorescence. Similar to previous reports in mouse ciliary mutants, opsin accumulation predates cell death, as the majority of PRs that displayed opsin accumulation were negative for TUNEL. However, we did observe TUNEL positive nuclei in a subset of PRs that had substantial amounts of intracellular opsins (Fig. 2H–I). We then raised a subset of *ta3−/−* larvae in constant darkness instead of the typical 14 hour light/10 hour dark cycle used in maintenance of zebrafish larvae. The amount of cell death seen in a TUNEL assay was significantly smaller in the retinae of larvae raised in darkness compared to those exposed to light, suggesting that intracellular opsin accumulation exerts a toxic effect after light exposure (Fig. 2G). Taken together, our results indicate that the observed intracellular opsin accumulation may account at least in part for the observed PR cell death, which increases after light exposure, in zebrafish *ta3* mutants.

### Loss of Ta3 leads to visual function loss

**Figure 3:**
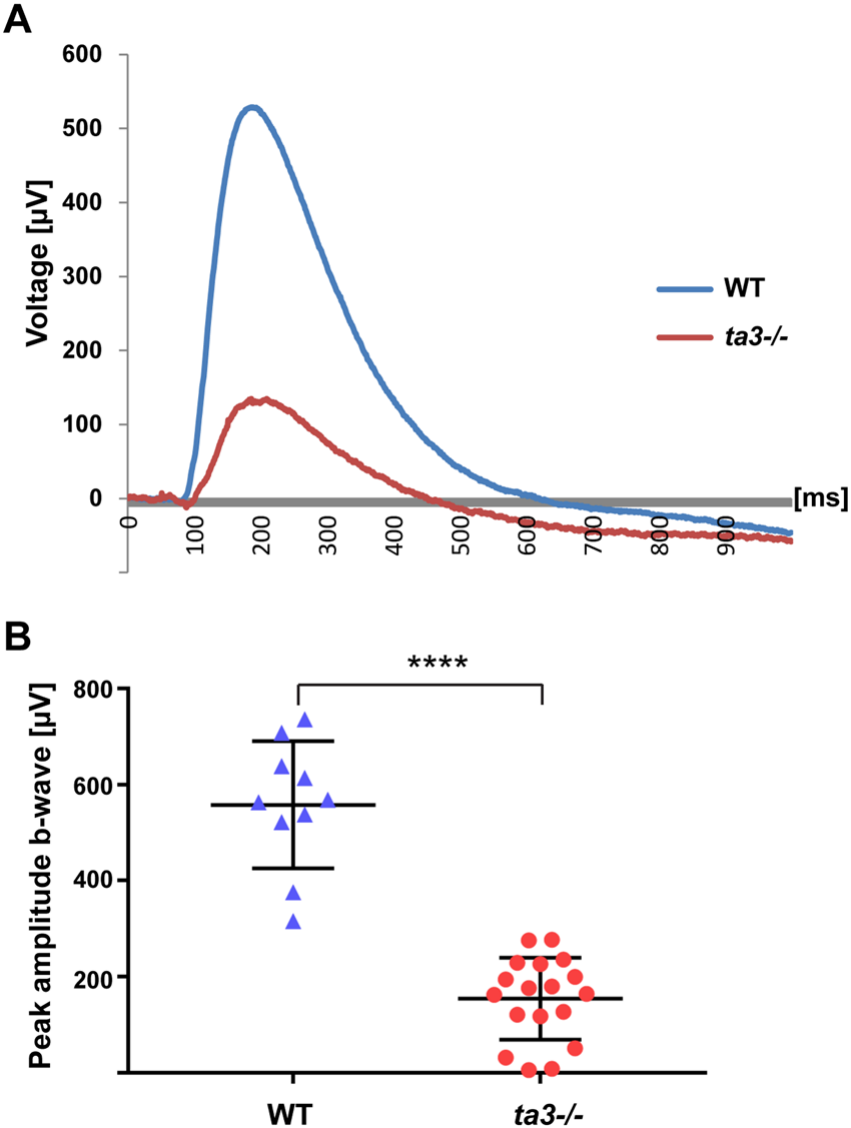
Loss of Ta3 leads to visual function loss. (**A**) Representative electroretinogram (ERG) recording of wildtype (WT, blue curve) and *ta3* mutant (red curve) 6 dpf larvae exposed to light stimuli of 7000 lux (bright light intensity). (**B**) Average b-wave peak responses for WT (blue triangles) and *ta3* mutants (red circles) (*n* = 18 *ta3* mutant animals and 10 WT animals). Bars represent standard deviation. ****p < 0.0001, Student’s *t*-test, *n* = 10 wildtype and 18 mutant larvae.

To assess the consequences of intracellular opsin accumulation and PR cell death on visual function, we performed electroretinograms (ERGs) at 6 dpf to determine the electrical response of the retina to bright light stimuli. The ERG response was highly significantly decreased in *ta3* mutants compared to wildtype (wt) even at the brightest light intensities (representative curves for wt and *ta3−/−* larvae in Fig. 3A, quantification in Fig. 3B; p < 0.0001, Student’s *t*-test). Therefore, the observed retinal PR degeneration and opsin mislocalization are associated with strongly decreased visual sensitivity in *ta3* mutant zebrafish.

### Deficient outer segment development in *ta3−/−* larvae

**Figure 4:**
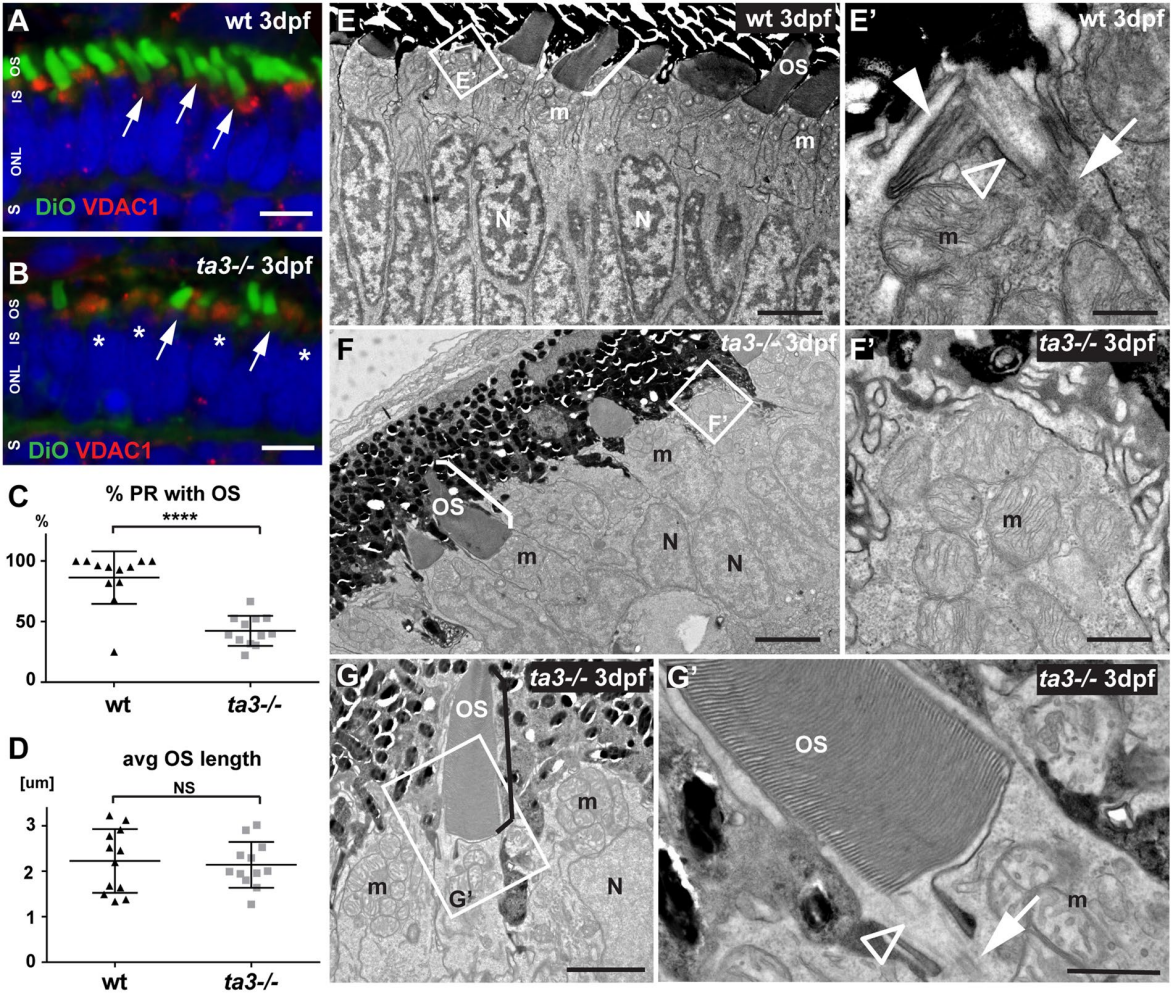
Deficient OS development in *ta3−/−* larvae in the majority of PRs. (**A**,**B**) 3 dpf cryosections stained with DiO to mark the OS (green, arrows) and with VDAC1 to mark mitochondrial clusters show reduced numbers of OSs in *ta3* mutants (**B**) compared to wildtype (wt) (**A**). Asterisks highlight PRs without OS in mutants. Nuclei are counterstained with DAPI (blue). (**C**) Quantification of the proportion of PRs with extended OSs in wt (black triangles) compared to *ta3* mutants (grey squares) shows significantly reduced PRs with OSs in mutants (****p < 0.0001*, t-test, n* = 12 wt and 12 mutant larvae). Each data point represents the proportion of PRs with OSs on a single confocal section of a single larva. Bars are standard deviation. (**D**) Quantification of average OS length on cryosections shows no significant difference between 3 dpf wt and *ta3* mutants (*p* = 0.7366, Student’s *t-*test, *n* = 12 wt and 12 mutant larvae). Each data point represents the average OS length on a single confocal section of a single larva. Error bars are standard deviation. (**E–G’**) Representative transmission electron microscopy images of 3 dpf wt (**E–E’**) and *ta3* mutant (**F–G’**) retinae. (**E–E’**) The majority of wt PRs have extended OSs (bracket in **E**) or are starting to stack membranes to do so (white arrowhead in **E’**). Note the BB (arrow in **E’**) and the connecting cilium (empty arrowhead in **E’**) in wt photoreceptors. (**F–F’**) In contrast, in *ta3−/−* PRs, only a minority of PRs have extended OSs (bracket in **F**), and no attempt at membrane stacking is observed in the other PRs (**F’**). (**G**) Note the normal appearance of the OSs that have extended in the mutants, including the structurally normal connecting cilium (arrowhead in **G’**) and basal body (arrow in **G’**). a*vg* average, *dpf* days post fertilization, *IS* inner segment, *m* mitochondria, *N* nuclei, *NS* not significant, *ONL* outer nuclear layer, *OS* outer segment, *PR* photoreceptor, *S* synapse, *wt* wild-type. Scale bars: 4 µm in (**A**,**B**), 3 µm in E and F, 0.5 µm in (**E’**), 2 µm in (**G**), 1 µm in (**F’**–**G’**).

Given that PR OSs are highly specialized ciliary compartments^8^, they are frequently structurally and/or functionally affected by mutations in ciliary genes. At 3 dpf, the majority of wildtype (wt) PRs have started developing OSs as seen on cryosections stained with the lipophilic dye DiO, which highlights the stacks of membranes composing the OSs (Fig. 4A). In *ta3* mutants however, we observed a significant reduction in the proportion of PRs forming OSs compared to wt (Fig. 4B; quantification in Fig. 4C, p < 0.0001, Student’s *t*-test, n = 12 wt and 12 *ta3* mutant larvae). However, those PRs that did extend OSs appeared indistinguishable from wt at 3 dpf: OSs were normally shaped and OS length was not different between wt and *ta3* mutants, as measured on 3 dpf cryosections (Fig. 4D). These findings were confirmed with transmission electron microscopy (TEM), showing that the majority of wt PRs had already elongated OSs at 3 dpf (Fig. 4E) and that those PRs that appeared to still lack OSs on cryosections had started the process of membrane stacking (Fig. 4E’). In contrast, no attempt at OS formation with membrane stacking was observed in the majority of *ta3−/−* PRs (Fig. 4F–F’). However, the OSs that did develop in *ta3* mutants had normal morphology (Fig. 4G–G’): The stacks of membrane disks were regularly organized and the basal body and connecting cilium indistinguishable from those seen in wt. Based on the immunofluorescence results presented in Fig. 1, it is most likely that persistance of maternally-deposited Ta3 allowed OS development in these PRs. Together, these results indicate that OS development in *ta3* mutants follows an “all or nothing” pattern: while the majority of PRs do not develop any OSs, those that do develop appear morphologically normal at 3 dpf.

### The deficient OS development of *ta3−/−* PRs is caused by abnormal BB localization

**Figure 5:**
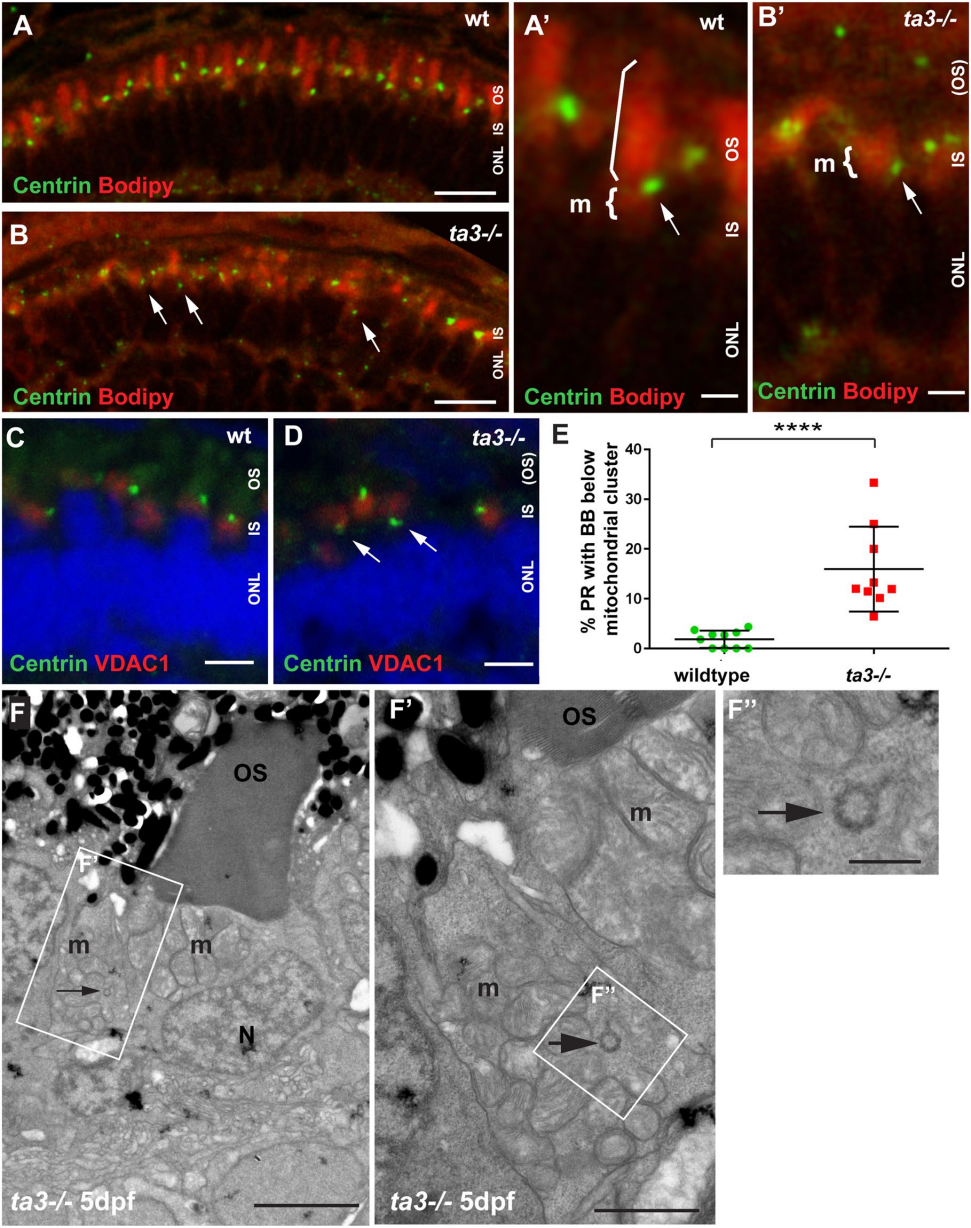
Abnormal BB localization in *ta3−/−* PR underlies the OS development defect. (**A**,**B**) 4 dpf cryosections stained with BODIPY (red) marking membranes of the OS and mitochondrial cluster and with anti-Centrin antibody (green) to mark the basal bodies, show aberrant localization of BB below the mitochondria in *ta3−/−* PRs. In wildtype (wt) PRs, the centrin-marked BB (arrow in **A’**) is located just basal to the OS (straight bracket) and apical to the mitochondrial cluster (curved bracket), while this localization is lost in a substantial number of *ta3* mutant PRs with BBs located below the mitochondrial cluster (arrow in **B’**). (**C**,**D**) Immunofluorescence on 4 dpf cryosections with anti-VDAC1 antibody (red) to mark the mitochondria and anti-Centrin antibody to mark the BB showing aberrant positioning of BBs in *ta3* mutants basal to the mitochondria (arrows in **D**) compared to wt (**C**). (**E**) Quantification of BB position with respect to the mitochondrial cluster based on immunofluorescence with anti-Centrin antibody. Each data point represents the proportion of PRs with BBs below the lower 1/3 of the mitochondrial cluster on one confocal section of a whole retina from one single larva. The proportion of 4 dpf PRs with aberrant BB positioning below the mitochondrial cluster is significantly increased in *ta3* mutants (****p < 0.0001, Student’s *t-*test, *n* = 10 wildtype and 9 mutant larvae). Bars are standard deviation. (**F–F”**) Representative TEM images showing presence of a BB in cross-section (arrow) within a mitochondrial cluster even at the later stage of 5 dpf, when all BBs should have docked to the apical membrane. (**F’**) Is the boxed area in (**F**) and (**F”**) is the boxed area in (**F’**). *BB* basal body, *IS* inner segment, *m* mitochondria, *N* nuclei, *ONL* outer nuclear layer, *OS* outer segment, *PR* photoreceptor. Scale bars: 10 µm in (**A**,**B**), 3 µm in (**A’**–**B’**), 4 µm in (**C**,**D**), 3 µm in (**F**), 1 µm in (**F’**) and 0.5 µm in (**F”**).

OS development requires docking of the BB and extension of the axoneme before stacking of membrane disks can occur^32^. Given the described role for Ta3 in early ciliogenesis, we evaluated if lack of OS development in *ta3−/−* PR was due to impaired BB docking. Indeed, while BBs marked with anti-Centrin antibodies were observed almost exclusively apically to the mitochondrial cluster and just below the BODIPY-marked OSs in wildtype (wt) retinae (Fig. 5A–A’ and C), we observed a significantly increased proportion of PRs with BBs located below the mitochondrial cluster in *ta3* mutants (Fig. 5B–B’,D and quantification in 5E; *p* < 0.0001, Student’s *t*-test, n = 10 wt and 9 *ta3* mutant larvae). These findings were further confirmed by transmission electron microscopy (TEM), which revealed BBs below and sometimes within the mitochondrial cluster in mutant PRs (Fig. 5F–F”). We additionally observed loss of the normally elongated PR cell shape in mutant PRs whose cell body took on a rounded shape with loss of inner segment space (Figs 2B’,5F, Figs S2A–H’ and S5). These results explain the lack of OS development in *ta3−/−* PRs through a role for Ta3 in BB docking in retinal PRs and suggest that Ta3 is also required for maintenance of PR cell shape.

### Outer segment development is rescued by constitutively active Rab8a

**Figure 6:**
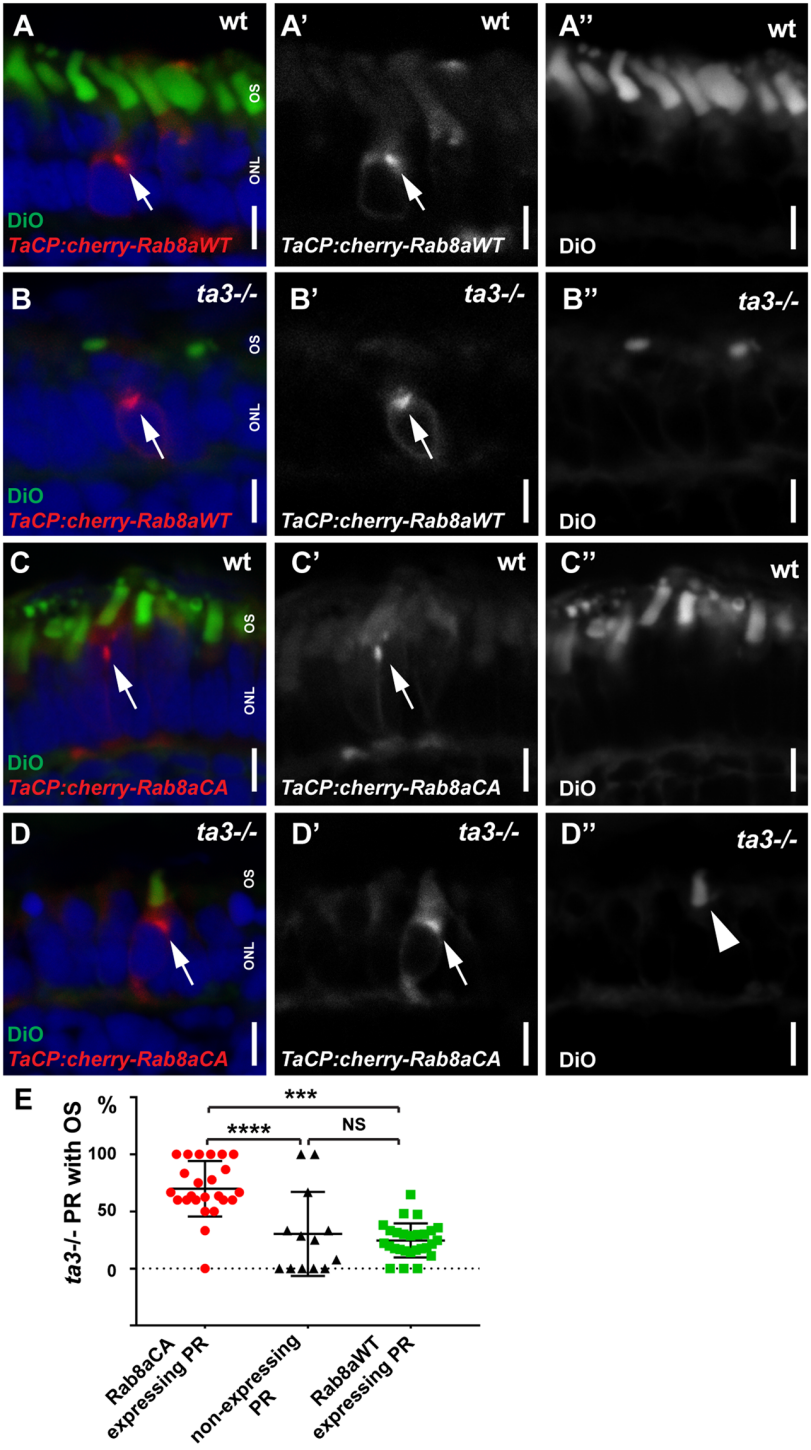
Outer segment development is rescued by constitutively active Rab8a. (**A–A”**) Cryosections of 4 dpf wt larvae with transient expression of WT mCherry-tagged Rab8a in cone PRs (*tacp:mCherry-Rab8aWT)* counterstained with DiO to mark OSs (green) and DAPI for nuclei. Note the punctate expression pattern of Rab8a concentrated in the inner segment space (arrow). (**B–B”**) A similar expression pattern is observed when expressing this transgene in *ta3−/−* PRs (arrow). (**C–C”**) Expression of a constitutively active form of mCherry-tagged Rab8a (*tacp:mCherry-Rab8aCA)* in wt cone PRs. (**D–D”**) *ta3−/−* PRs expressing constitutively active Rab8a develop OSs (arrowhead in D”). (**E**) Quantification of the proportion of 4 dpf *ta3* mutant PRs expressing constitutively active Rab8a with OSs (red circles), *ta3* mutant PRs expressing wild-type Rab8a with OSs (green squares) or *ta3* mutant PRs without transgenic Rab8a expression with OSs (black triangles). Only the constitutively active form of Rab8a rescues OS formation in *ta3* mutants (****p < 0.0001, NS not significant, Student’s *t-*test, *n* > 20 larvae expressing either transgene and 13 non-transgenic larvae). Each datapoint represents one individual larva. Bars are standard deviation. *ONL* outer nuclear layer, *OS* outer segment, *S* synapse. Scale bars: 3 µm in all panels.

Initial stages of ciliogenesis require docking of the ciliary vesicle onto the mother centriole, a process in part controlled by the small GTPase Rab8^22^. After docking of the BB at the apical membrane, ciliary membrane biogenesis depends on Rab8 as well^33^. To test whether loss of Ta3 in PRs affects Rab8 localization, as described in RPE1 cells, we transiently expressed an mCherry-tagged version of Rab8a in cone PRs (*taCP:mCherry-Rab8aWT*)^34^. We chose to focus on cones since this PR type is largely predominant in 4 dpf zebrafish retinae, while rods are not contributing functionnally to the visual response at this stage^35^. Moreover, we recently showed that Rab8 trafficking occurs similarly in rods and cones based on live imaging of tagged Rab8 in zebrafish PRs^36^. We observed no difference in expression pattern between wildtype and *ta3−/−* PRs (Fig. 6A–B”): mCherry expression was concentrated in a punctate manner in the inner segment in expressing wt and *ta3−/−* PRs. Moreover, expression of mCherry-tagged Rab8 had no effect on the presence or absence of OS in these cells (Fig. 6E, green squares). However, when we transiently expressed a constitutively active form of Rab8a, using the same promoter (t*aCP:mCherry-Rab8aCA*), we observed highly significant rescue of OS development in the mutant PRs expressing this transgene (Fig. 6D–D’ and red circles in E, p < 0.0001, Student’s *t*-test), with a majority of expressing PRs now displaying a nicely elongated OS. Similar results were obtained when overexpressing the constitutively active form of Rab8a in rod PRs (Fig. S6). This implies that BBs had docked properly and ciliogenesis had occurred normally in *ta3−/−* PRs expressing an active Rab8a form and indicates that the role of Ta3 in early ciliogenesis is upstream of Rab8a activation.

## Discussion

*KIAA0586/TA3* mutations cause a wide range of ciliopathies in humans, ranging from the milder Joubert syndrome^10,11,23,37^, which presents with retinal dystrophy in 30% of individuals^15^, to syndromes associated with fetal lethality such as hydrolethalus syndrome or short-rib polydactyly^16^. Our analysis using a zebrafish *ta3* mutant is the first model for Ta3-related retinal dystrophy, identifying a role for Ta3 in the development and function of retinal PRs. Our findings confirm that the previously ascribed function of Ta3 in BB positioning and docking^19–21^ is important in PRs and indicate that this function is upstream of Rab8a activation, since OS extension can be rescued by a constitutively active form of this small GTPase.

Previous work in cell culture suggested a model in which Ta3 regulates the timely dispersal of centriolar satellites prior to cilium formation, which in turn affects Rab8 localization and/or activation during cilia formation^22^. Our results are consistent with this model, placing Ta3 function in PRs upstream of Rab8a activation in ciliogenesis. However, in contrast to work in cell culture, we did not observe significantly altered WT-Rab8 localization in zebrafish PRs in our experiments. This discrepancy may be explained by differences in methodology: during cilium extension, which can be visualized as it proceeds in cultured cells after serum starvation, Rab8 enters the ciliary compartment in control RPE1 but not in TA3 knock-down cells^22,38,39^. After the cilium has extended, Rab8 leaves the ciliary compartment of wildtype cells^39^. In whole zebrafish larvae, cilium extension cannot be induced and thus followed dynamically and therefore, since the cilium is already extended, Rab8 is not seen within the ciliary compartment (OS) but mainly concentrated in the inner segment, where it is involved in additional cellular processes beyond initial ciliogenesis, including ciliary-directed trafficking of opsin carrier vesicles in photoreceptors^36,40,41^. Our results indicate that activated Rab8 can find its proper subcellular localization to perform its function in ciliogenesis in PRs even in the absence of Ta3. One role for Ta3 may lie in localizing Rab8 activators such as Rabin8^33^ or RPGR^42^ to the BB, possibly through additional indirect steps given the lack of identification of a direct interaction between Ta3 and Rabin8^22^. Following this hypothesis, Rab8 could not be activated in the absence of Ta3, leading to the observed defects in ciliogenesis, absence of OS formation and secondary intracellular accumulation of opsins.

Mislocalization of opsins to the cell body appears to be at least in part responsible for the observed cell death in zebrafish *talpid3−/−* PRs. As intracellular opsin accumulation mostly preceeds cell death, this indicates that this mislocalization is not a secondary effect in already degenerating PRs, similar to what has been shown in mouse ciliary mutants^43,44^. Rather, opsins likely cannot be properly targeted in the absence of an outer segment and accumulate in the cell. Moreover, the occasional co-occurrence of TUNEL positive signal in cells that also have major intracellular opsin accumulations suggests that cell death follows opsin mislocalization relatively rapidly. Finally, while mouse studies have not found a deleterious effect of light exposure on PR survival, our data indicate that fish exposed to light for 14 hours a day do show increased cell death compared to fish raised in constant darkness. Differences in life cycle (diurnal zebrafish vs nocturnal mice) may explain these discrepant results. Indeed, since mice are mostly active during dark and inactive during light hours, the difference in light exposure between mice kept on a light cycle vs those kept in constant darkness might be smaller than the difference between light and dark-raised zebrafish, as zebrafish will be active during the 14 hours of daylight. Experiments in which mice carrying mutations in ciliary genes are raised in constant very bright light conditions could test this hypothesis.

In addition to the intracellular accumulation of opsins and the lack of OS formation, we also observed progressive loss of PR cell shape with displacement of the mitochondrial cluster next to the nucleus instead of its normal apical position, suggesting a loss in cytoskeletal organization or cell polarity. A role for Ta3 in cell polarity was previously suggested based on experiments in the chick embryo, in which Golgi apparatus localization was affected by Ta3 loss-of-function^23^. Since the BB also serves as microtubule organization center for the cell, in addition to its role in nucleating the axoneme of the primary cilium, its aberrant localization likely leads to disorganization of the cytoskeleton. Intriguingly, we observed that even *ta3−/−* PRs that had developed an initially normal-looking OS, likely thanks to rescued BB docking through maternally-deposited Ta3, displayed progressive loss of normal cell shape. This suggests that Ta3 may be required for maintenance of cell shape even after the BB has docked and the axoneme extended.

Our findings are further consistent with recent work which identified TA3 as an interaction partner of the microtubule actin crosslinking factor 1 (MACF1), whose deficiency leads to similar PR phenotypes in mouse retina as we observe in zebrafish^45^. Indeed, mice lacking MACF1 function display deficient ciliogenesis due to lack of BB migration and docking as well as defects in OS maintenance in adult PRs after conditional knock-out. Moreover, defects in PR cell polarity are observed in *Macf1* mutants, leading the authors to propose that TA3 is involved in the coordination of microtubule and actin interactions. Our findings are consistent with this model in which Ta3 may play a role as microtubule-organization center at the BB after it has docked to the apical membrane. Thus, Ta3 may play distinct roles in initial ciliogenesis and in maintenance of cytoskeletal integrity after ciliogenesis has occurred, the former lying upstream of Rab8 activation, while the latter may involve regulation of microtubule-actin interactions.

Further work will be required to investigate this model and unravel additional potential functions for Ta3 beyond BB docking, which may be of prime importance for understanding and treating human disease caused by mutations in KIAA0586*/TA3*. Indeed, while a major ciliogenesis defect may underlie the more severe human ciliopathies caused by mutations in this gene, the milder phenotypes of Joubert syndrome may be caused by more subtle ciliary dysfunction, which may in turn be more amenable to specific therapies.

## Material and Methods

### Zebrafish

Zebrafish *(Danio rerio)* were maintained as described^46^. Embryos were raised at 28 °C in embryo medium and pigment development was inhibited by phenylthiourea for immunohistochemistry as described in Westerfield^46^. The *talpid3* ^*i262, i263, i264*^ mutants (referred to as *ta3* mutant or *ta3−/−*) were previously described^24^. Likewise, the *ift88*^tz288^ *oval* mutant was previously published^27,28,47^. The *tg(taCP:mCherry-Rab8a)* construct was previously described^34,48^. Site targeted mutagenesis using overlapping complementary primers was employed to generate the Q67L (CAG to CTG codon) point mutation leading to constitutively active Rab8a^41,49^. The primer sequences used were (modified nucleotides are indicated in lower case): 5′-CCGCAATACGTTCAGTATG-3′ and 5′-CCGAAATCGTTCCaGTC-3′ to amplify from the 5′UTR to the mutation site, and 5′-CAGGACtGGAACGATTTC-3′ and 5′-GAGAGATGGGATAAAAGAGG-3′ to amplify from the mutation site to the 3′UTR. cDNA obtained from whole zebrafish larvae at 5 dpf was used as template. Gateway® (Invitrogen) recombination using the Tol2kit^50^ was performed to generate the *taCP:mCherry-Rab8aCA* and *rhod:mCherry-Rab8aCA* constructs which were co-injected with Tol2 transposase as previously described^51^ into 1-cell stage offspring of incrossed *ta3*+/− zebrafish.

### Ethics statement

All animal protocols were in compliance with internationally recognized and with Swiss legal ethical guidelines for the use of fish in biomedical research and experiments were approved by the local authorities (Veterinäramt Zürich Tierhaltungsnummer 150).

### Immunohistochemistry, TUNEL cell death assay and light microscopy

Zebrafish larvae were fixed in 4% PFA overnight at 4 °C, embedded in OCT and cryosectioned following standard protocols. For Ta3 antibody staining, zebrafish larvae were anesthesized with tricaine, washed unfixed in 30% sucrose for 30 minutes and embedded in OCT for cryosectioning. Only sections of equivalent regions of the eyes were analyzed (determined by size and shape of the lens and/or the optic nerve). For immunofluorescence, the sections were blocked using PBDT (PBS, 1% DMSO, 0.5% Triton X-100, 2 mg/ml BSA) with 10% goat serum for 30 minutes at RT before incubation with primary antibodies overnight. Primary antibodies were mouse monoclonal anti-acetylated Tubulin (1:500, Sigma T6793), mouse anti-Centrin (1:200, clone 20H5 Millipore), mouse anti-Opsin 4D2 and 1D1 (1:100, gift from R. Molday, University of British Columbia), zpr1 (1:100, ZIRC), VDAC1 (1:100 abcam, ab15895). Secondary antibodies were Alexa Fluor goat anti-rabbit or goat anti-mouse IgG (Life Technologies) used at 1:400. BODIPY® TR Methyl Ester (1:300, Invitrogen) or Vybrant®-DiO cell labeling solution (1:200, Invitrogen) were applied for 20 minutes and nuclei were counterstained with DAPI. TUNEL assay was performed following manufacturer protocol (*In Situ* Cell Death Detection Kit, Fluorescein, Roche). In brief, cryosections were incubated 20 minutes in permeabilization solution, then 2 hours in TUNEL solution at 37 °C. All immunofluorescence experiments were performed at least in duplicate (independent clutches and/or independent days) with at least 10 animals per condition (the number of animals is visible in the graphs as each datapoint is one animal). Confocal imaging was performed on a Leica HCS LSI or a Leica SP5 microscope.

### Transmission electron microscopy

For Transmission Electron Microsopy, larvae were fixed overnight at 4 °C in a freshly prepared mixture of 2.5% glutaraldehyde and 2% paraformaldehyde in 0.1 M sodium cacodylate buffer (pH 7.4). After rinsing in buffer, specimens were washed in 1% osmium tetroxide in 0.1 M sodium cacodylate buffer (pH 7.4), during 2 h at room temperature. After rinsing, tissues were dehydrated through a graded series of ethanol ranging from 50% to 100% and embedded in Epon. Ultrathin sections (70 nm) comprising zebrafish eyes were obtained using a Leica Ultracut UCT ultramicrotome and collected on formvar coated grids and examined with a Philips CM-100 scope. Images were acquired using the Gatan Microscopy Software.

### Ta3 antibody generation

Custom polyclonal peptide antibodies were raised by Eurogentec (Seraing, Belgium) using their standard 87-day protocol. Rabbits were immunized with the Ta3 peptide H2N-CRR LSD DAF FGA DEK GED T-CONH2. Antibodies were affinity-purified against the corresponding peptide.

### Electroretinography (ERG)

Normal (white) ERG was recorded on 6 dpf larvae as previously described^52^. Briefly, larvae were dark adapted for at least 30 min, the eye ball was isolated and placed in the middle of the recording chamber which was filled with 1.5% agarose. The recording electrode was positioned against the central cornea while the reference electrode was inserted into the recording chamber and underneath the eye. Each larva was submitted to three bright light stimuli (7000 lux) with an inter stimulus interval of 10 seconds. The average of the 3 responses was calculated.

### Quantifications and statistics

All quantifications were performed on whole retinal cryosections: confocal stacks of identical thickness or single confocal sections of equivalent regions of the eyes for all conditions were analysed. For quantification of cell death using the TUNEL assay, all DAPI-marked nuclei that had positive TUNEL-fluorescence were counted over the entire 5 µm-thick stack of whole retinal sections. For quantification of the proportion of PRs with OS and for OS length, all DiO-highlighted OSs on equivalent whole retinal sections were analysed. The proportion of PRs with OSs was determined by dividing the number of DiO-marked OSs through the number of PR nuclei visible on that section. OS length was determined by measuring the longest distance of the fluorescent DiO signal in the apico-basal axis of the PR cell. For quantification of BB position, all centrin-marked basal bodies on single confocal sections of entire retinae were analyzed; the position of the BB was judged to be “basal to the mitochondrial cluster” in a given PR if the centrin fluorescence was below (=basal to) the lower 1/3 of the mitochondrial cluster. For quantification of OS rescue through constitutively active Rab8 in cones, all the PRs expressing the transgene in a given whole retinal section were analyzed and presence or absence of the DiO-marked OS in those PRs was determined. Proportion of non-expressing cells with OSs was determined by counting the number of DAPI-stained nuclei and the number of OSs in cells without *tacp:mCherry-Rab8a* expression (given that the vast majority of PRs are cones at this developmental stage). For assessment of rescue through *rhod:mCherry-Rab8a* in rods, the rod PRs were additionally marked with the 1D1 antibody and only nuclei of cells positive for 1D1 staining were considered. For all quantifications, data were pooled from at least 2–3 independent experiments (independent clutches/independent experiment days). All quantification data used to generate the graphs are found in the supplementary xls spreadsheet. Graphpad Prism software was used for statistical analysis and generation of plots. Unpaired t-test was applied for comparisons between wild-type and mutants.

### Data availability

All data generated or analysed during this study are included in this published article (and its Supplementary Information files).

## Electronic supplementary material

Supplementary information

Quantification data

## Electronic supplementary material

**Supplementary information** accompanies this paper at 10.1038/s41598-018-20489-9 .
