# Publisher Correction: The ciliopathy protein TALPID3/KIAA0586 acts upstream of Rab8 activation in zebrafish photoreceptor outer segment formation and maintenance

**DOI:** 10.1038/s41598-018-30671-8

**Published:** 2018-08-17

**Authors:** Irene Ojeda Naharros, Flavia B. Cristian, Jingjing Zang, Matthias Gesemann, Philip W. Ingham, Stephan C. F. Neuhauss, Ruxandra Bachmann-Gagescu

**Affiliations:** 1 Institute for Molecular Life Sciences, University of Zurich, 8057 Zurich, Switzerland; 2 Lee Kong Chian School of Medicine, Nanyang Technological University, 639798 Singapore, Singapore; 3 Institute for Medical Genetics, University of Zurich, 8952 Schlieren, Switzerland; 4 Present Address: Department of Human Molecular Genetics, Institute of Human Genetics, University of Heidelberg, Heidelberg, Germany

Correction to: *Scientific Reports* 10.1038/s41598-018-20489-9, published online 02 Febraury 2018

In the original version of this Article, the author Irene Ojeda Naharros was incorrectly indexed. This error has now been corrected.

