# Supplementary material for: The ciliopathy protein TALPID3/KIAA0586 acts upstream of Rab8 activation in zebrafish photoreceptor outer segment formation and maintenance
Source: Sci Rep. 2018 Feb 2;8:2211. doi: 10.1038/s41598-018-20489-9 (PMC5797153; doi:10.1038/s41598-018-20489-9)
Supplement: Supplementary file 1 — Supplementary information [file 41598_2018_20489_MOESM1_ESM.pdf]

The ciliopathy protein TALPID3/KIAA0586 acts upstream of Rab8 activation in zebrafish photoreceptor outer segment formation and maintenance

Irene Ojeda Naharros[1], Flavia B. Cristian[1,*], Jingjing Zang[1], Matthias Gesemann[1], Philip Ingham[2], Stephan C.F. Neuhauss[1], Ruxandra Bachmann-Gagescu[1,3]

1 Institute for Molecular Life Sciences, University of Zurich, 8059 Zurich, Switzerland

2 Lee Kong Chian School of Medicine, Nanyang Technological University, 639798 Singapore, Singapore

3 Institute for Medical Genetics, University of Zurich, 8952 Schlieren, Switzerland

* Current address: Department of Human Molecular Genetics, Institute of Human Genetics, University of Heidelberg, Germany

Supplementary information

Figure S1: Ta3 protein localization time course in PRs

Figure S2: Time course of retinal degeneration in *ta3*-/- zebrafish

Figure S3: Differentiating *ta3-/-* PRs take on cone- and rod-specific cell fates

Figure S4: PR cell death in *ta3* mutant retinae

Figure S5: Progressive cell shape loss of *ta3*-/- PRs

Figure S6: Outer segment development is rescued by constitutively active Rab8a in rods

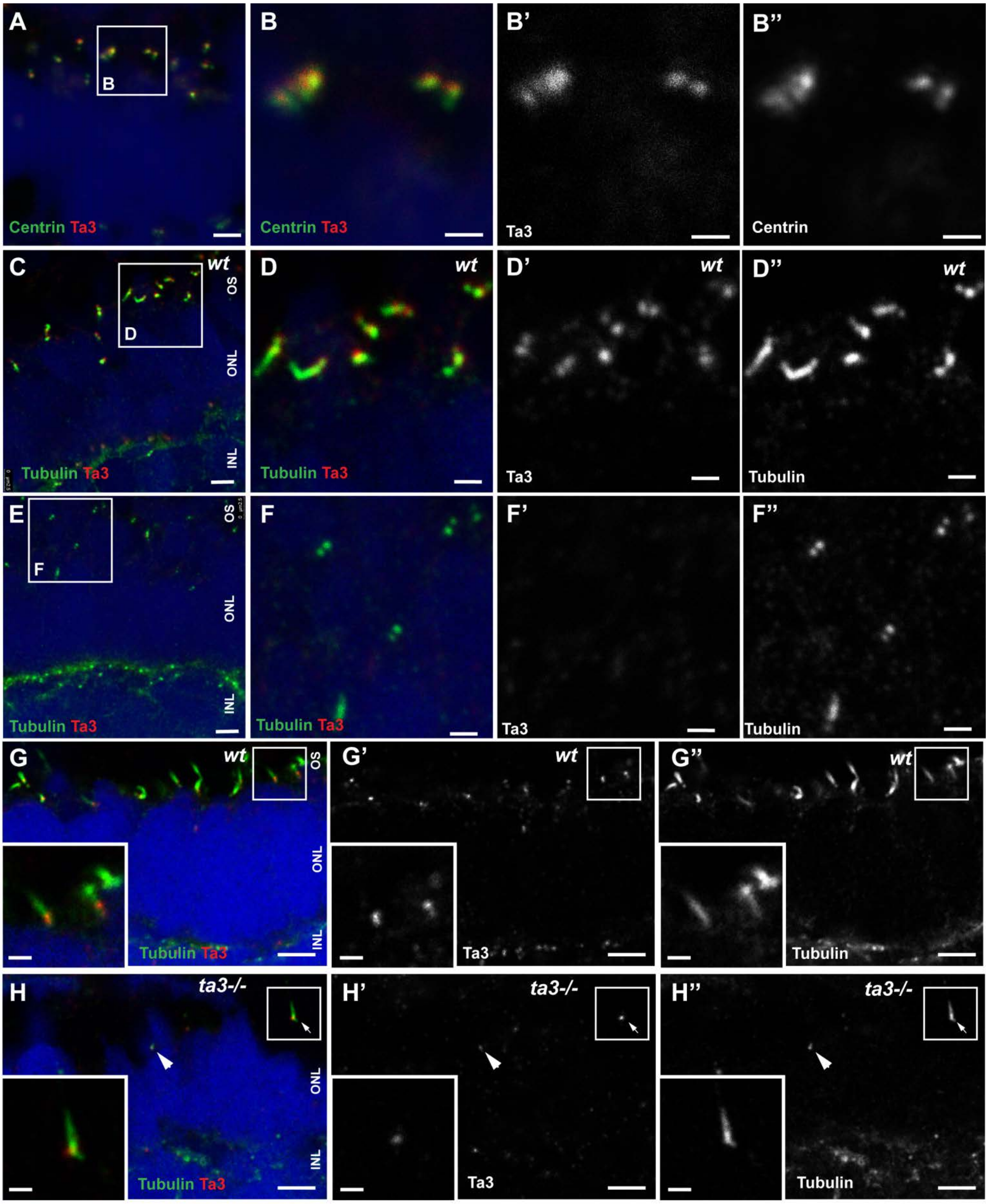

Figure S1: Ta3 protein localization time course in PRs
(A-B'') 2 dpf retinal cryosections stained with anti-Centrin to mark BBs (green) and anti-Ta3 (red) show localization of Ta3 protein at the BB in wildtype (wt) larvae. **(C-D'')** 3 dpf cryosections of wt retinas stained with anti-acetylated tubulin and anti-Ta3

show localization of Ta3 at both mother and daughter centrioles. **(E-F'')** In contrast, Ta3 signal is abolished from the majority of BBs in 3dpf *ta3* mutants. **(G-G'')** 4 dpf cryosections of wt retinas stained with anti-acetylated tubulin and anti-Ta3 show similar results as at 3 dpf. **(H-H'')** In *ta3*-/- PRs at 4 dpf, the Ta3 signal is mostly abolished **(F'')** except in a few isolated PRs (arrowhead and arrow in **H-H'**). Note that the two PRs still expressing Ta3 are also the only ones to have an extended axoneme (arrowhead and arrow in **F''**). The boxed area in **A** is shown in **B-B''**, the one in **C** is shown in **D-D''** and the one in **E** is shown in **F-F''**. The insets in **G-H''** represent the boxed areas in the corresponding images. Scale bars: 2.5 µm in (A and C), 1 µm in (B-B'', D-D'' and insets in E-F''), 4 µm in (E-F'').

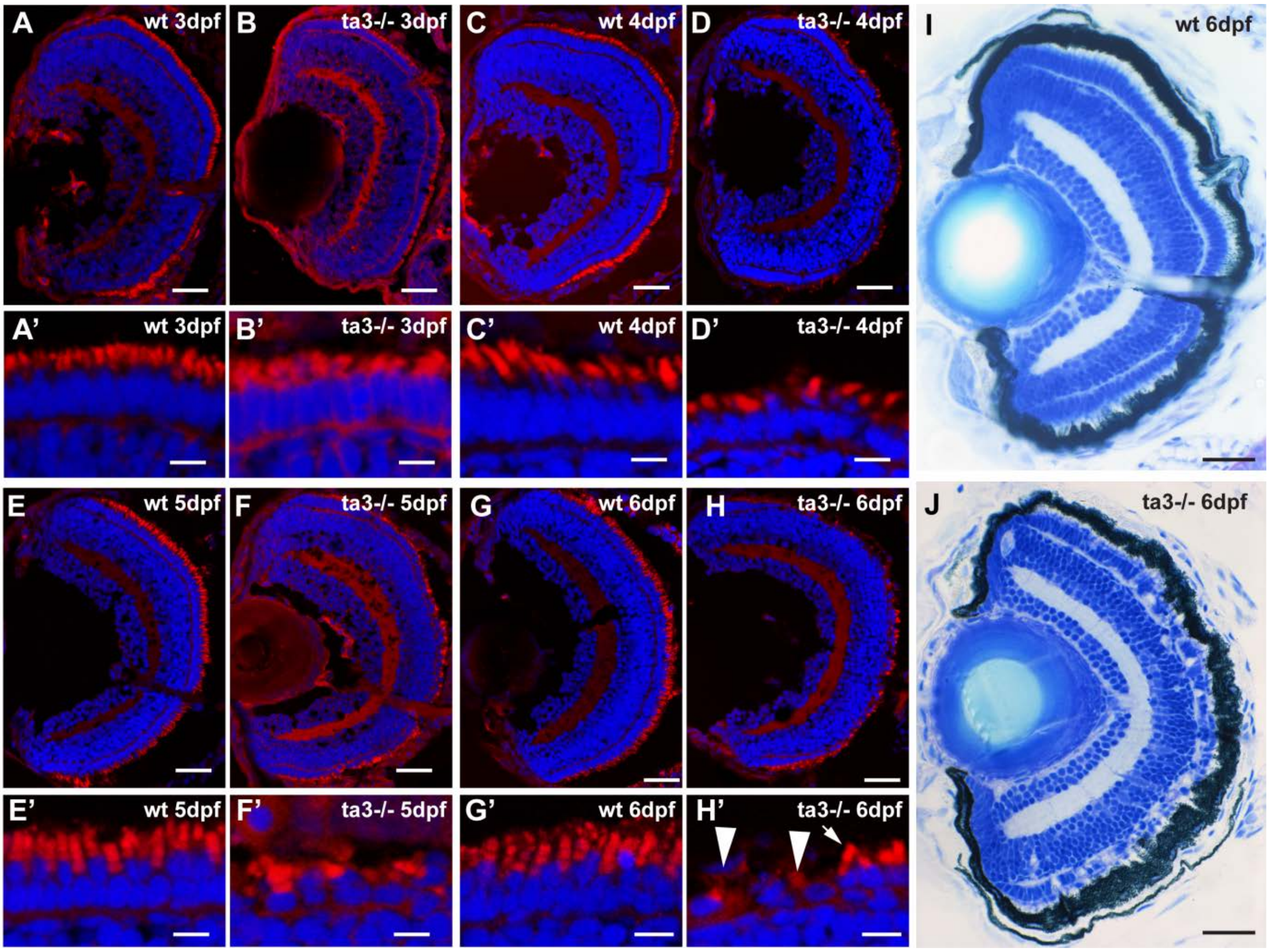

Figure S2: Time course of PR degeneration in *ta3* mutants
Retinal cryosections of wt and *ta3*-/- zebrafish at 3dpf **(A-B')**, 4dpf **(C-D')**, 5dpf **(E-F')** and 6 dpf **(G-H')**. Nuclei are counterstained with DAPI and membranes (including outer segments) are highlighted with BODIPY. Note the progressive cell shape loss and decreased numbers of outer segments in mutants. The arrow in H' indicates a PR with an OS, while the arrowheads point to PRs without OSs. **(I-J)** Histological sections through whole wt and *ta3*-/- eyes at 6dpf showing gaps in the PR cell layer, but also persistance of many PR cells. Scale bars: 30 µm in (A-H) and (I-J), 4 µm in A'-H'.

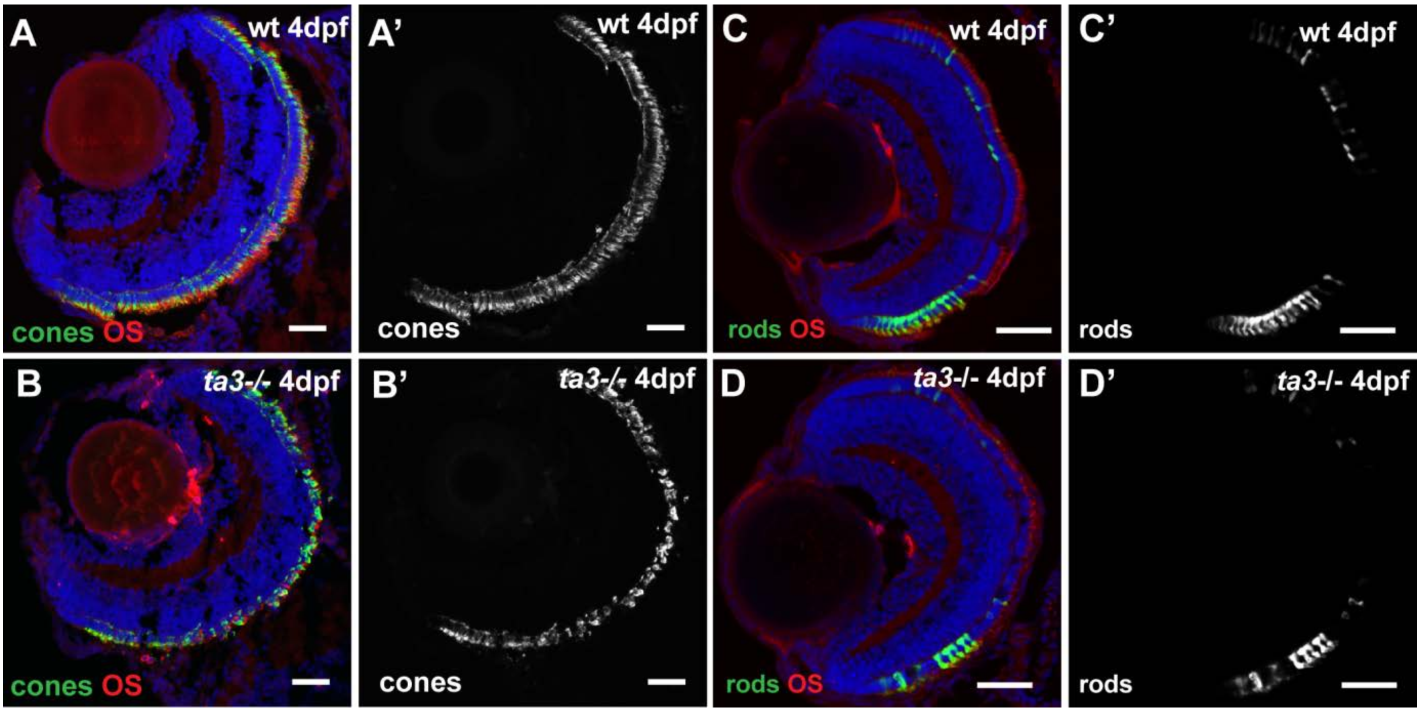

Figure S3: Differentiating *ta3-/-* PRs take on cone and rod specific cell fates (A-B) Retinal cryosections of 4 dpf wt and *ta3*-/- larvae stained with the zpr1 antibody indicating that mutant photoreceptors take on a cone-specific cell fate. **(C-D')** Likewise, *ta3*-/- photoreceptors take on a rod-specific fate as seen with the transgenic line *tg(zfRH1-3.7B:EGFP)* marking rod photoreceptors (PRs), seen here in retinal cryosections of 4dpf wt (C-C') and *ta3* mutant (D-D') fish. Scale bars: 30 µm in all panels.

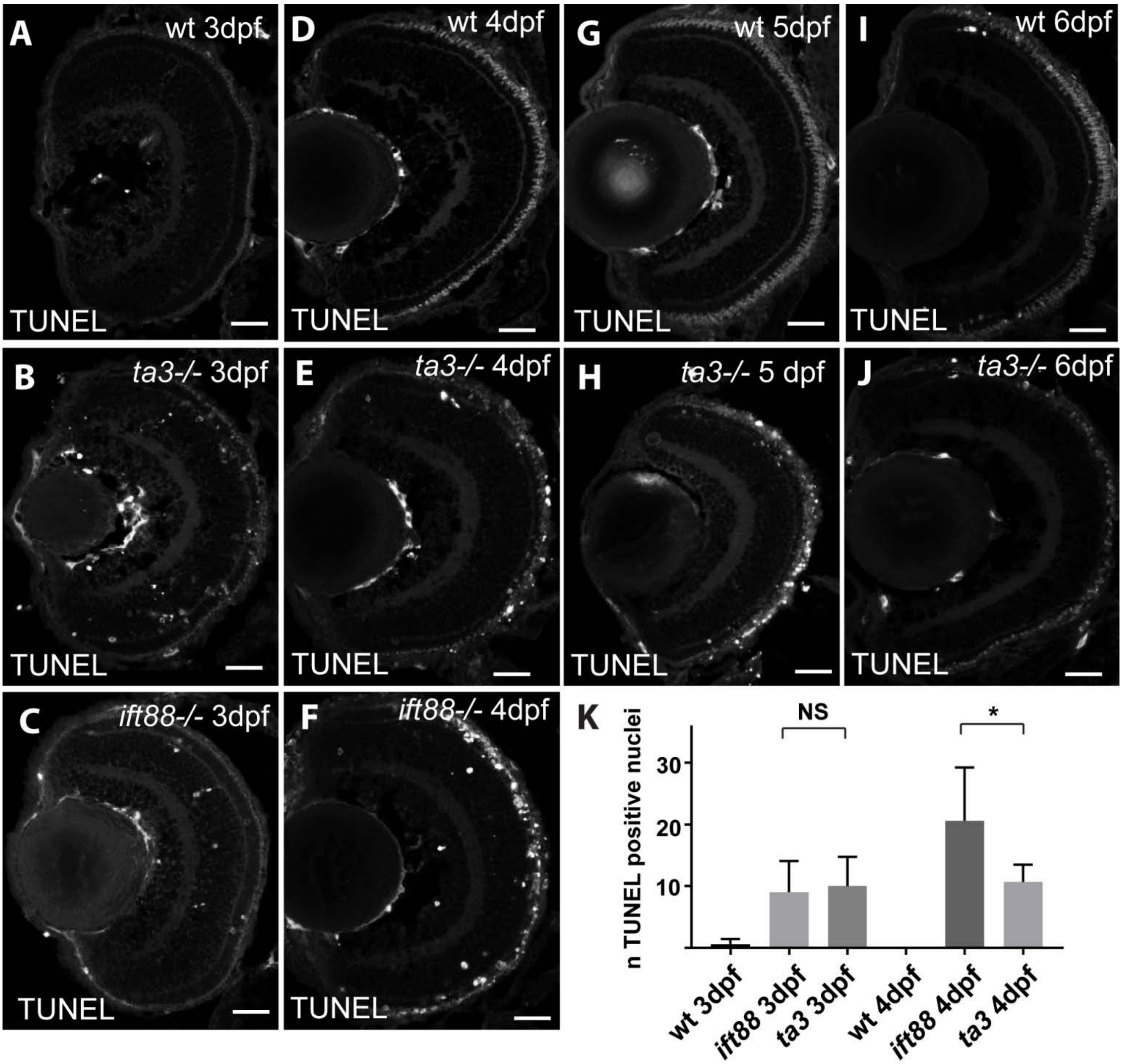

Figure S4: PR cell death in *ta3* mutant retinae

TUNEL staining on retinal cryosections at 3 dpf **(A-C)**, 4 dpf **(D-F)**, 5 dpf **(G-H)** and 6 dpf **(I-J)**, in wildtype **(A,D,G** and **I)**, *talpid3* mutants **(B,E,H** and **J)** and *ift88* mutants **(C** and **F)**. Note the strong signal in *ift88* mutants at 4 dpf and the comparatively lower amount of cell death in *ta3* mutants at the same stage. **(K)** Quantification of cell death in wt, *ift88* and *ta3* mutants at 3 and 4dpf. The number of TUNEL-positive nuclei was counted on 5 µm-thick confocal sections of entire retinal sections. Note the stable rate of cell death in *ta3* mutants between 3 and 4dpf while *ift88* mutants show an increase in cell death at 4dpf compared to 3dpf. Scale bars: 20 µm in all panels.

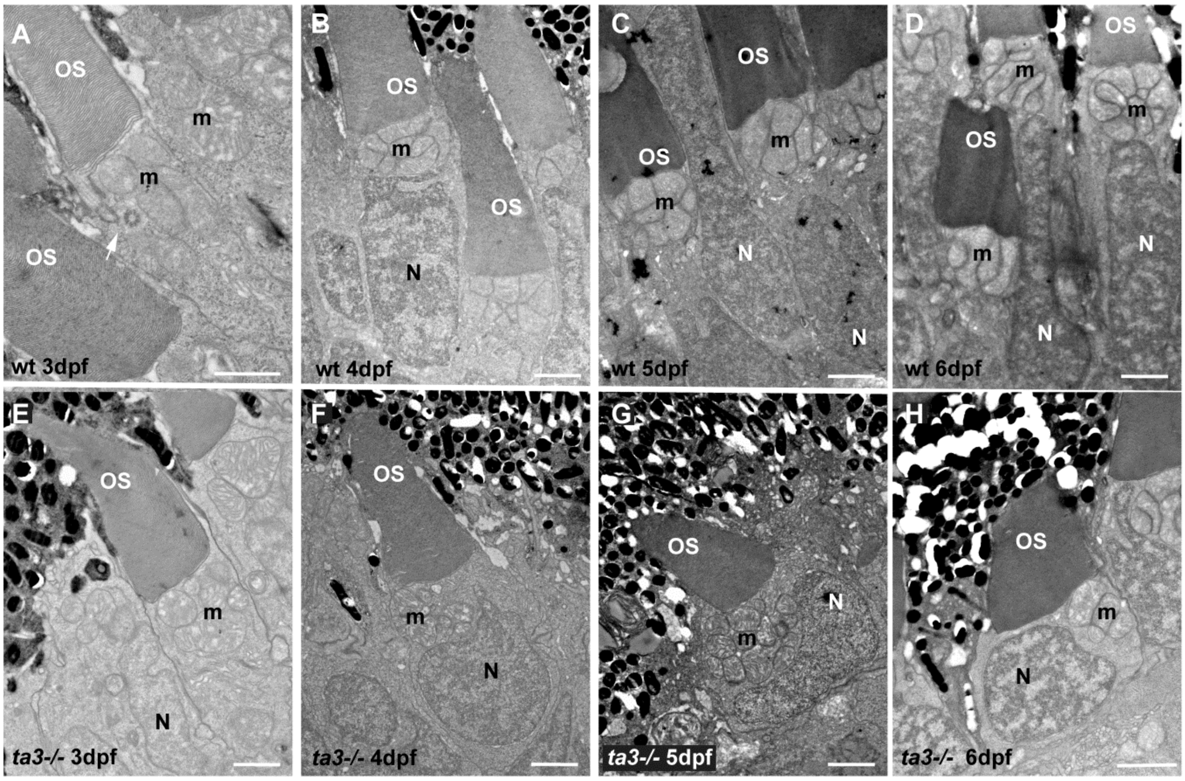

Figure S5: Progressive cell shape loss of *ta3*-/- PRs
Transmission electron microscopy images of wt **(A-D)** and *ta3-/-* **(E-H)** PRs showing the progressive collapse of the normally apico-basally elongated cell shape of PRs. At 6 dpf, note the lack if inner segment space and the mitochondrial location next to the nucleus instead of its normal apical position **(H).**

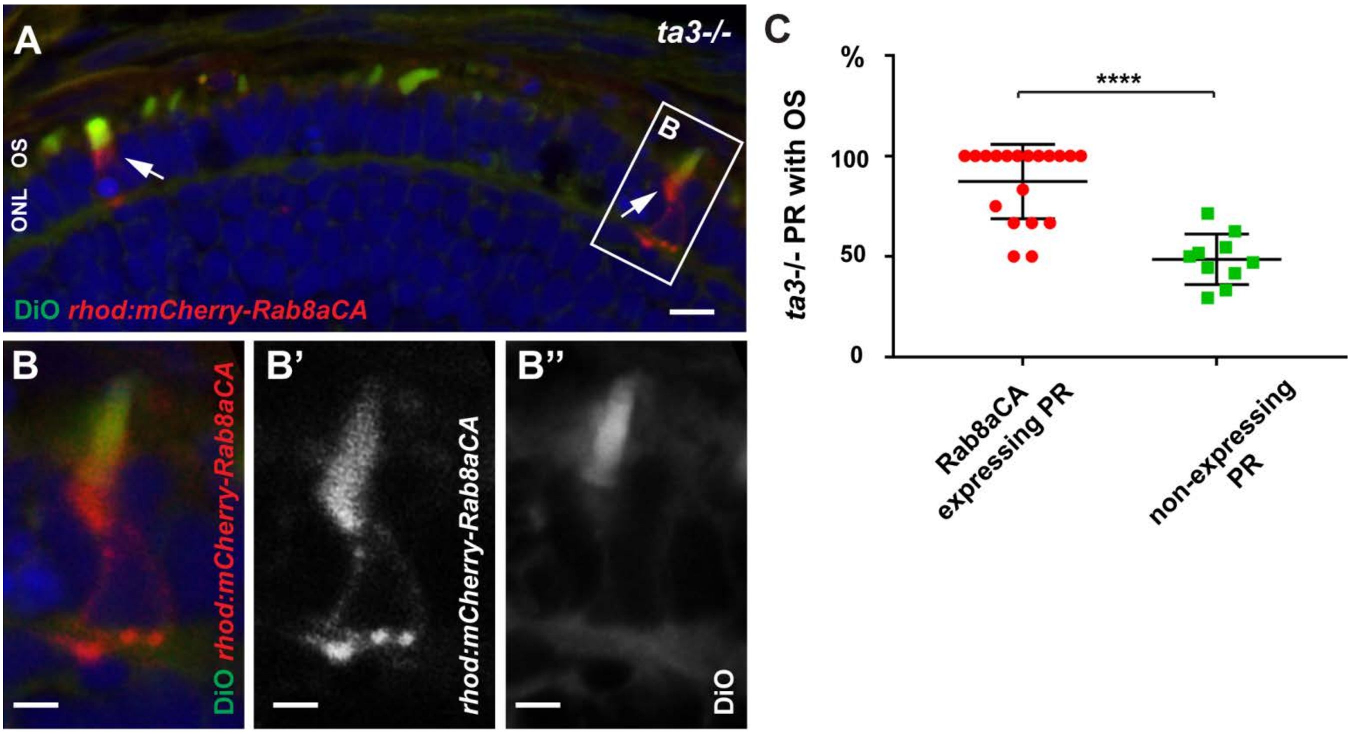

Figure S6: Rescue of OS development by expression of constitutively active Rab8a in rod PRs

(A) Cryosection of 4 dpf zebrafish *ta3*-/- larva expressing constitutively active Rab8a in rod PRs (*rhod:mCherry-Rab8aCA*, red) and stained with DiO (green) to highlight membranes and OSs. Rod PRs expressing Rab8aCA develop OSs. **(B)** is a close-up image of the inset in (A). **(B')** shows only the red channel (Rab8aCA) and **(B'')** shows the green channel highlighting the presence of the OS. Scale bars are 4 µm in A and 2 µm in B-B''. **(C)** Quantification of OS development in rod PRs expressing Rab8aCA. Each red datapoint (circles) represents the percentage of PRs expressing the transgene that developed an OS in a single larva, while each green datapoint (squares) indicates the percentage of rod PRs ot expressing the transgene that developed OSs.
